# Intercellular cooperation in a fungal plant pathogen facilitates host colonization

**DOI:** 10.1073/pnas.1811267116

**Published:** 2019-02-06

**Authors:** Rémi Peyraud, Malick Mbengue, Adelin Barbacci, Sylvain Raffaele

**Affiliations:** ^a^Laboratoire des Interactions Plantes-Microorganismes (LIPM), Institut National de la Recherche Agronomique (INRA), CNRS, Université de Toulouse, 31326 Castanet-Tolosan, France

**Keywords:** division of labor, fungi, systems biology, virulence, metabolic heterogeneity

## Abstract

Cooperation is associated with major transitions in evolution such as the emergence of multicellularity. It is central to the evolution of many complex traits in nature, including growth and virulence in pathogenic bacteria. Whether cells of multicellular parasites function cooperatively during infection remains, however, largely unknown. Here, we show that hyphal cells of the fungal pathogen *Sclerotinia sclerotiorum* reprogram toward division of labor to facilitate the colonization of host plants. Using global transcriptome sequencing, we reveal that gene expression patterns diverge markedly in cells at the center and apex of hyphae during *Arabidopsis thaliana* colonization compared with in vitro growth. We reconstructed a genome-scale metabolic model for *S. sclerotiorum* and used flux balance analysis to demonstrate metabolic heterogeneity supporting division of labor between hyphal cells. Accordingly, continuity between the central and apical compartments of invasive hyphae was required for optimal growth *in planta*. Using a multicell model of fungal hyphae, we show that this cooperative functioning enhances fungal growth predominantly during host colonization. Our work identifies cooperation in fungal hyphae as a mechanism emerging at the multicellular level to support host colonization and virulence.

## Significance

Cooperation between specialized cells and organisms supports complex biological functions, from the colonization of unfavorable environments to the formation of organs and sociality. Some bacterial pathogens are known to rely on cooperation between individuals and species for efficient colonization of their host and the onset of disease. We examined the regulation of genes in cells from different parts of a fungal plant pathogen and found evidence for cooperation between these fungal cells. We further show that cooperation between fungal cells is particularly important for the efficient colonization of resistant plants. These findings establish cooperation as a mechanism supporting disease caused by fungal pathogens that should be taken into account in the design of disease management strategies.

Cooperation is the process by which biological units, or modules, incur a cost to provide a benefit to the group they belong to (1). The emergence of cooperation typically is associated with division of labor—the specialization of several modules (cells, organs, or whole organisms), each devoted to specific tasks. Cooperation also generally involves the exchange of molecules between specialized modules, a process called “resource allocation” in the case of cytoplasmic metabolite exchange between cells (2–5). The efficiency with which a group of cells exploits resources of their environment may increase as a consequence of cooperation. For instance, in some syntrophic microbial communities, the secretion of digestive enzymes by individual specialized cells changes local resource availability, which in turn can benefit neighboring cells (6, 7). Cooperation is associated with major transitions in the evolution of life through the emergence of new functions at a higher level of organization, such as multicellularity and sociality (2, 8). It is the basis of many complex traits in nature, including virulence in some pathogenic species. In bacterial pathogens, cooperative behaviors such as biofilm formation, swarming motility, and the secretion of virulence factors are crucial to virulence and controlled by quorum sensing, the release and perception of small signal molecules (9–11). Distinct developmental and functional stages, interdependent for nutrition, were documented in the mycelium of wood-decay fungi in response to local environmental changes within host tissues (12–14). In the fungal meningitis pathogen *Cryptococcus gatii*, cooperation has also been associated with the emergence of outbreak strains that proliferate rapidly within host cells (15). Imaging and modeling approaches emphasized the importance of intrahyphal nutrient translocation for growth in Ascomycete and Basidiomycete fungi (14, 16). However, since the formulation of the cell theory in the 1830s, the eukaryotic cell has often been considered as an autonomous life unit (17). A number of processes such as the circadian clock, embryonic cell fate determination, and immunity can indeed be cell-autonomous in multicellular organisms (18). Furthermore, disorders such as cancers and neurodegenerative diseases result from the proliferation of one cell type detrimental to the whole organism (4). The extent to which cooperation contributes to complex traits, such as host colonization in pathogens with diverse infection strategies, remains largely unknown.

Natural selection shapes genes, cells, and organisms to promote their own evolutionary success at the expense of their competitors. In this context, the emergence of cooperation is favored only when specific conditions are met (8). Genetic proximity between individuals, also designated as kin selection, is a well-established condition that increases the likelihood of cooperative phenotypes to evolve (1). Cooperation is also expected to prevail if cooperative modules are segregated in space and interact preferentially with specific neighbors (19, 20). Spatial game theory suggests that the spatial structure of interacting populations can promote cooperation (21). Experiments in synthetic yeast colonies showed that spatial expansion promotes the evolution of cooperation locally (22). Variation, in the form of mutations, phenotypic changes, or local environment properties, is another factor favoring task specialization and cooperation (23). For instance, the random and progressive introduction of metabolic auxotrophies in yeast populations leads to the spontaneous establishment of metabolically cooperating communities (5).

The white mold fungus *Sclerotinia sclerotiorum* has a devastating impact on crops such as rapeseed and soybean, threatening food security worldwide (24). Like other fungal pathogens, it derives energy from its hosts for growth and reproduction. To do so, it produces a mycelium consisting of interconnected linear hyphae that colonize host tissues intercellularly and secrete proteins and metabolites that modify host cells’ physiology (25, 26). *S. sclerotiorum* has a necrotrophic lifestyle and rapidly causes host cell death and host tissue maceration at the center of the infected area, while the margin of the mycelium spreads through living plant cells (27). The morphology of *S. sclerotiorum* hyphae also differs between the margin and the center of the colony during plant infection (28). These hyphae are exposed to a heterogeneous and variable environment, with the apex of the mycelium growing through intact host tissues while its center is surrounded by a host-derived substrate with distinct chemical and physical properties. *S. sclerotiorum* therefore presents several properties favorable to the establishment of cooperative phenotypes that prompted us to test whether virulence is associated with intercellular cooperation. Here we show that compartments of *S. sclerotiorum* invasive hyphae cooperate through resource allocation and division of labor to facilitate host colonization, supporting a role for intercellular cooperation in fungal virulence.

## Results

### Transcriptome Analyses Reveal the Spatial Organization of *S. sclerotiorum* Invasive Hyphae.

**Fig. 1.**
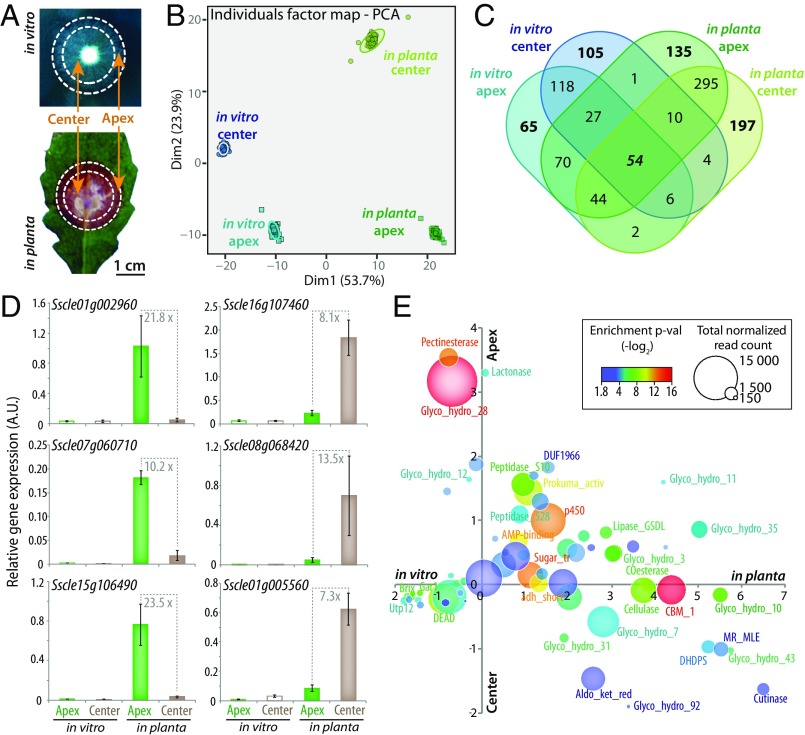
Global transcriptome analyses reveal the spatial organization of fungal cells *in planta*. (*A*) Sampling strategy for the analysis of spatial differentiation of *S. sclerotiorum* global transcriptome in vitro and *in planta* on *A. thaliana* leaves. (*B*) Individual factor map of a PCA on *S. sclerotiorum* top differentially expressed genes. Ellipses show 95% confidence interval for each sample, calculated based on shuffling gene expression 100 times between three biological replicates. (*C*) Venn diagram showing the distribution of genes induced at least fourfold compared with in vitro liquid medium growth condition. (*D*) Quantitative RT-PCR validation of the expression profile for three genes specifically expressed at the mycelium apex *in planta* (plain green boxes) and three genes specifically expressed at the center of mycelium *in planta* (plain brown boxes). Error bars show SD of the mean for three biological replicates. (*E*) Enrichment analysis for PFAM domain annotations among differentially expressed genes in each sample type. The *x* axis shows enrichment in vitro versus *in planta* and the *y* axis shows enrichment in mycelium center versus apex. Annotations are colored according to the minimum enrichment *P* value (*P* values < 0.018 are shown), and sized according to the cumulated number of normalized sequence reads.

To test experimentally for spatial organization in *S. sclerotiorum* invasive hyphae, we analyzed the global transcriptome of *S. sclerotiorum* mycelium central and apical areas by RNA sequencing (RNA-seq). We harvested in triplicate separate areas corresponding to the central and apical zones of fungal mycelium grown on solid medium in vitro and on *Arabidopsis thaliana* plants (Fig. 1*A*). The first two principal components of the global gene expression dataset accounted for ∼77.6% of the total variation in read count (Fig. 1*B*). The major driver of differential gene expression was growth conditions (in vitro versus *in planta*, accounting for 53.7% of total variance), followed by the mycelium area (central versus apical, accounting for 23.9% of the total variance); these effects greatly exceeded the effects from biological replicates. The amplitude of principal component 2, which separates samples based on colony areas, was largely determined by the samples collected *in planta*, suggesting that the transcriptional differentiation of mycelium center and apex was stronger *in planta*. We used gene expression in *S. sclerotiorum* grown in liquid medium determined by RNA-seq as a reference to determine gene induction folds. We identified a total of 1,133 genes (10.2% of the genome) induced fourfold either *in planta* or on solid medium in vitro (Fig. 1*C* and Dataset S1). Only 54 genes (4.7%) were induced in all four conditions, and 218 genes (19.2%) were induced both in vitro and *in planta*. A total of 288 genes (25.5%) were induced in vitro only, and 627 (55.4%) were induced *in planta* only, indicating that plant colonization requires extensive specific transcriptional reprogramming. A total of 553 genes (48.8%) were induced both at the center and at the apex of colonies, indicating that a majority of transcriptional reprogramming is area-specific. Among genes induced in the mycelium center only (306 in total), there were only four (1.3%) induced both in vitro and *in planta*, further illustrating specific and local transcriptional reprogramming of *S. sclerotiorum* during *A. thaliana* colonization.

To confirm patterns of local cellular specialization in *S. sclerotiorum* during plant colonization, we analyzed the expression pattern of selected genes by quantitative RT-PCR. We found that the expression of some fungal genes was restricted to cells of the apex or the center of the mycelium (Fig. 1*D*). The *Sscle01g002960* peptidase and the *Sscle15g106490* and *Sscle07g060710* oxidoreductases showed, respectively, a 21.8-, 10.2-, and 23.5-fold higher expression at the apex of *S. sclerotiorum* mycelium *in planta* than in any other tested condition. Conversely, the *Sscle16g107460* aldo-keto reductase, the *Sscle08g068420* CO-esterase, and the *Sscle01g005560* aminotransferase showed, respectively, 8.1-, 13.5-, and 7.3-fold higher expression at the center of *S. sclerotiorum* mycelium *in planta* than in any other tested condition. This validates the specific and local activation of some *S. sclerotiorum* genes during host colonization. To document the nature of specific and local transcriptional programs, we grouped induced genes according to their predicted function. We analyzed in which condition these functions were predominantly activated by testing for enrichment *in planta* compared with in vitro and at the margin compared with the center of the mycelium (Fig. 1*E*). We found that a number of key metabolic functions for host colonization were performed locally. Functions strongly enriched at the apex of the mycelium include pectinesterases and polygalacturonases (GH28) that degrade pectic homogalacturonans, β-1,4-endoglucanases (GH12) and β-1,4-xylanases (GH11), serine proteases (peptidases S10, S28, and prokumamolysin), and cytochrome p450, notably involved in fungal toxin biosynthesis (29) (*SI Appendix*, Fig. S1). Functions strongly enriched at the center of the mycelium include arabinofuranosidases, β-1,4-xylanases (GH43), and α-1,4-galactosidase that cleave carbohydrate backbones, cutinases, muconate lactonizing enzyme (MR_MLE) involved in the breakdown and assimilation of aromatic compounds, and dihydrodipicolinate synthase (DHDPS), a key enzyme in lysine biosynthesis. We conclude that host colonization triggers division of labor in *S. sclerotiorum* invasive hyphae with specific gene expression patterns at the apex and the center of the mycelium.

### Division of Labor Drives Metabolic Heterogeneity Along *S. sclerotiorum* Invasive Hyphae.

**Fig. 2.**
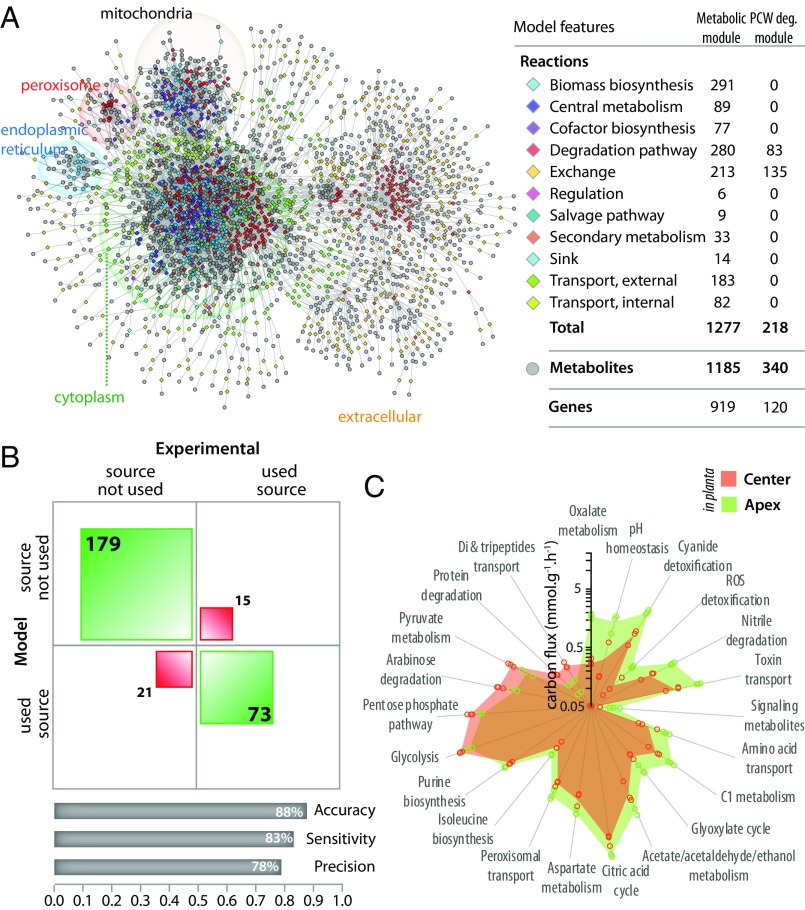
Division of labor drives metabolic heterogeneity along *S. sclerotiorum* hyphae during host colonization. (*A*) Overview of reactions and metabolites included in *S. sclerotiorum* GEM highlighting the major cellular compartments and the extracellular compartment. The table describes features of *S. sclerotiorum* GEM with the number of reactions and genes associated with the metabolic module and plant cell wall (PCW) degradation modules. (*B*) Experimental evaluation of the performances of *S. sclerotiorum* GEM using Biolog phenotype microarrays. (*B*, *Top*) Contingency table of the modeled (rows) and experimentally determined (columns) metabolic capacities for *S. sclerotiorum*. The squares are sized according to the number or carbon sources; correct predictions are shown in green and incorrect predictions in red. (*B*, *Bottom*) Bars show performance statistics. (*C*) Radar plots showing metabolic pathways supporting significantly different carbon fluxes in center and apex cells during *A. thaliana* colonization. Shaded areas show mean fluxes calculated by FBA on three biologically independent global transcriptome sequencings, with values for individual replicates shown as dots.

To assess quantitatively the impact of division of labor on major cell functions in *S. sclerotiorum* hyphae, we performed a flux balance analysis (FBA) using experimentally determined transcriptomes. To this end, we first reconstructed a genome-scale metabolic model (GEM) of *S. sclerotiorum* (Fig. 2*A* and Dataset S2). Our model includes a metabolic module with 1,277 reactions of the central metabolism, nutrient uptake pathways, degradation pathways, and the biosynthesis pathways of the major components of the fungal biomass. The model also includes a plant cell-wall degradation module with 218 reactions catalyzed by secreted plant cell-wall-degrading enzymes. Altogether, *S. sclerotiorum* GEM encompasses 1,495 unique reactions associated with 1,039 genes, comparable to validated yeast and filamentous fungi models (30, 31). To evaluate the performance of this GEM, we determined experimentally *S. sclerotiorum* growth on 288 metabolites using Biolog phenotype microarrays and compared it with model predictions (Fig. 2*B* and Dataset S3). The model correctly predicted the observed behavior on 252 metabolites (73 used as a C or N source and 179 not used), reaching 88% accuracy, 83% sensitivity, and 78% precision for axenic growth.

Next, we used FlexFluxOmics (32) to assess metabolic fluxes through *S. sclerotiorum* GEM based on global gene expression determined experimentally in vitro and *in planta*. We normalized flux distributions by fixing the biomass production fluxes measured experimentally for each condition. In *S. sclerotiorum* cells growing in vitro, 536 to 555 reactions supported nonnull fluxes, covering 79 to 86 distinct metabolic pathways. During *A. thaliana* colonization, 592 to 610 reactions supported nonnull fluxes in *S. sclerotiorum* cells, covering 101 to 103 distinct metabolic pathways (Dataset S4). Among these pathways, 16 supported significantly higher fluxes in apex compared with center cells during *A. thaliana* colonization, including detoxification pathways (cyanide, reactive oxygen species, and nitrile detoxification), biosynthesis of organic acids (oxalate and acetate metabolism and pH homeostasis), carbohydrate anabolism (glyoxylate and citric acid cycles and C1 metabolism), and secondary metabolite trafficking (peroxisomal and toxin transport) (Fig. 2*C*). Conversely, six pathways supported significantly lower fluxes in apex compared with center cells during *A. thaliana* colonization, related to energy and reduced cofactor production (glycolysis and pentose phosphate pathways with the respective upstream pathways, pyruvate metabolism, and arabinose degradation). By contrast, there was no pathway supporting significantly higher fluxes in apex compared with center cells during in vitro growth. This observation is consistent with apex and center cells performing complementary metabolic functions during plant colonization. In this analysis, predictions for pathways known to be regulated posttranscriptionally should be taken with caution as these regulation events are not included in the model. For instance, we identified higher fluxes through glycogen metabolism in center cells, but we could not determine whether biosynthesis or degradation of glycogen was activated since these pathways are controlled by phosphorylation events. To further characterize metabolic heterogeneity, we calculated total carbon metabolic uptake and carbon secretion for major virulence functions (oxalic acid production and protein secretion) by *S. sclerotiorum* apex and center cells (Fig. 2*D*). During in vitro growth, carbon metabolic uptake was similar in apex and center cells (20.0 ± 0.5 and 20.4 ± 1.9 C mmol⋅g^−1^⋅h^−1^, respectively, *P* = 0.73), as well as carbon secretion for virulence functions (0.80 ± 0.05 and 0.67 ± 0.35 C mmol⋅g^−1^⋅h^−1^ in apex and center cells, respectively, *P* = 0.56) (Dataset S4). The total carbon metabolic uptake was also similar in apex and center cells during plant colonization (19.9 ± 0.7 and 18.4 ± 3.4 C mmol⋅g^−1^⋅h^−1^, respectively, *P* = 0.52). However, the flux of carbon secretion for virulence was 8.5-fold lower in center cells (0.34 ± 0.06 C mmol⋅g^−1^⋅h^−1^) than in apex cells (2.89 ± 0.34 C mmol⋅g^−1^⋅h^−1^, *P* = 0.0047), suggesting that center cells may store carbon and transfer it toward apex cells, as observed in some filamentous fungi (14, 16, 33, 34). Metabolic exchange between center and apex cells prompted us to test whether the continuity between hyphal cells is required for successful host colonization.

### Continuity Between Central and Apical Compartments of *S. sclerotiorum* Hyphae Facilitates Host Colonization.

**Fig. 3.**
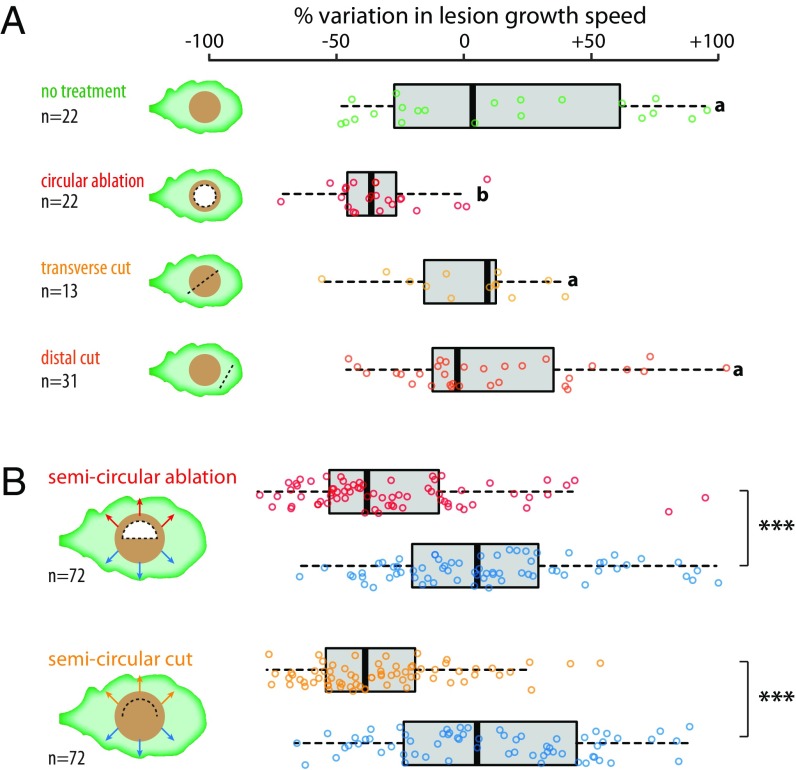
Optimal growth of *S. sclerotiorum* during *A. thaliana* colonization requires central hyphal compartments. (*A*) Variation in the speed of lesion radial growth between 13 and 17 hpi by *S. sclerotiorum*, as a percent of lesion growth speed between 9 and 13 hpi. An average 38% decrease in lesion growth was measured upon circular ablation of the lesion center at 13 hpi (red). Lesion growth was mostly unaffected in untouched lesions (green), after transverse cut (yellow), and after distal cut (orange). Significance groups were determined by pairwise Welch *t* tests (*P* < 0.01). (*B*) Variation in the speed of lesion radial growth between 13 and 17 hpi by *S. sclerotiorum* measured outward of semicircular ablation of the lesion center (red), semicircular cut (yellow), and on the symmetric untouched half of the same lesions (blue; see leaf diagrams). Significant differences between groups were determined by pairwise Welch *t* tests (***P < 0.01).

Molecules such as glucose can be transported over long distance in fungal hyphae (33, 34). After the ablation of subapical hyphal compartments, the growing hyphal tip is rapidly isolated from damaged cells by Woronin bodies and continues to grow in isolation (35, 36). To test whether the continuity between hyphal cells plays a role in host colonization, we inoculated *A. thaliana* host plants with *S. sclerotiorum* and measured the hyphal linear growth *in planta* (Fig. 3*A*). The average growth rate was 0.37 mm⋅h^−1^ between 9 and 13 h postinoculation (hpi). In untouched disease lesions, the hyphal growth rate remained unchanged between 13 and 17 hpi. When we performed at 13 hpi a circular ablation of the center of the lesion (and fungal hyphae it contains), the hyphal growth rate decreased on average by 38% (Student’s *t* test, *P* = 2.7e^−05^). To exclude the negative effect of a damage signal from the plant or the fungus, we performed transverse cuts across the disease lesion and cuts in distal parts of the leaf. This did not alter hyphal linear growth rate (Student’s *t* test, *P* = 0.19 and 0.45, respectively). Next, we performed semicircular ablations and semicircular cuts at 13 hpi and compared hyphal radial growth on the treated and untreated halves of the colony (Fig. 3*B* and *SI Appendix*, Fig. S2). Growth in the untouched halves of the lesions remained unaffected overall. Lesion growth was reduced by ∼28.7% (*P* = 1.8e^−07^) downstream of semicircular ablations and reduced by ∼33.3% (*P* = 9.1e^−11^) downstream of cuts. Thus, disruption of hyphal continuity only reduced the growth of cells connected radially. We conclude that continuity between the central and apical compartments, required for resource allocation among hyphal cells, increased *S. sclerotiorum* hyphal growth *in planta* and thereby facilitated host colonization.

### Division of Labor and Resource Allocation Increase Fungal Invasive Growth Synergistically.

**Fig. 4.**
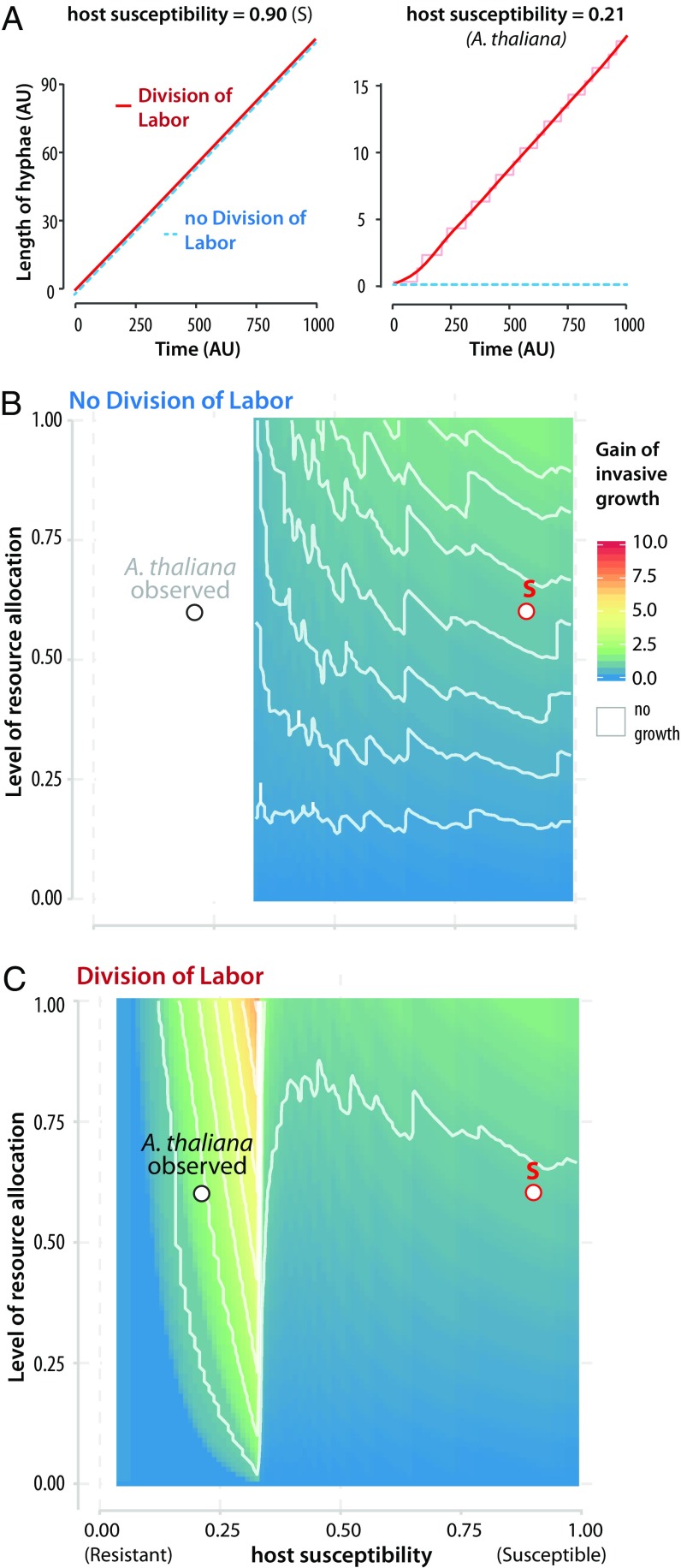
Division of labor and resource allocation increase fungal invasive growth on resistant hosts in a multicell model. (*A*) Simulated fungal invasive growth (*y* axis) on hosts with contrasted susceptibility levels over 1,000 iterations of the model (Time, *x* axis). Simulations were run with division of labor (DOL, red line) and without DOL (blue dotted line) on a susceptible host (*Left*, level of susceptibility = 0.9) and a resistant host (*Right*, level of susceptibility = 0.21). Suppression of DOL abolished fungal growth on a host of susceptibility 0.21, which corresponds to observed susceptibility for *A. thaliana* Col-0. AU, arbitrary units. (*B*) Gain of fungal invasive growth (color scale) in the absence of DOL, defined as the ratio between the number of hyphal cells reached after 1,000 iterations of the model with and without resource allocation, according to the level of resource allocation (*y* axis) and the level of host susceptibility (*x* axis). Curves of identical gain are shown as white lines. The values of parameters estimated experimentally for *S. sclerotiorum* during the colonization of *A. thaliana* are labeled on the graph. The point S corresponds to parameters used for the simulation with host susceptibility 0.90 shown in *A*. No fungal growth is predicted in white sectors of the graphs, suggesting that *A. thaliana* Col-0 colonization is not possible without DOL. (*C*) Gain of fungal invasive growth (color scale) with DOL according to the level of resource allocation and the level of host susceptibility. DOL enabled the colonization of host with susceptibility <0.3 such as *A. thaliana* Col-0. Predicted growth gains are higher on resistant hosts (susceptibility between 0.1 and 0.3) than on susceptible hosts (green-yellow sector).

Previous work reported a role for multicellularity in fungal growth in vitro (37). Our results demonstrate that intercellular cooperation in *S. sclerotiorum* hyphae promotes host colonization. To estimate the relative impact of intercellular cooperation on *S. sclerotiorum* fitness in various environments, we modeled invasive growth as a proxy for fitness (38, 39) in simulations with environments of various resistance levels. For this, we developed a biophysical multicell model of the growing hyphae during host infection. Following observations on fungal model systems (40, 41), we modeled the hyphae as a stack of cells with a single dividing apical cell (Fig. 4*A*). In light of our FBA results, we considered that energetic costs associated with the production of virulence factors decreased upon the death of the host cells, corresponding to the ability of the fungus to overcome host resistance (detoxification of host environment, degradation of host-derived compounds, and host manipulation). The diffusion of metabolites through the hyphae tends to minimize the gradient of metabolites between cells, according to the second law of thermodynamics. We considered the difference of energetic costs associated with virulence factors in apical and central cells of the hyphae as a measure of the degree of division of labor, and a proxy for the relative level of host susceptibility (from 0.0 fully resistant to 1.0 fully susceptible). We considered the rate of cytoplasmic diffusion between hyphal cells as a proxy for the degree of resource allocation among hyphal cells. The model allowed simulating hyphal growth according to (*i*) the relative susceptibility of host compartments, (*ii*) the degree of resource allocation between hyphal cells, and (*iii*) division of labor in the hyphae. We simulated hyphal length (Fig. 4*A*, *y* axis) in hosts with contrasted susceptibility levels between 0 and 1,000 iterations of the model (*x* axis) to monitor invasive growth. On a susceptible host (susceptibility 0.9; Fig. 4*A*, *Left*), the virtual hyphae grows rapidly, reaching a length >100 in 1,000 iterations. There is no apparent impact of suppressing division of labor in the model (dotted blue line). When host susceptibility is lower (0.21; Fig. 4*A*, *Right*), hyphal growth is reduced, reaching a length of 18 in 1,000 iterations, and can be completely suppressed in the absence of division of labor (dotted blue line).

To explore the effect of resource allocation and division of labor on hyphal growth in various environmental conditions, we calculated the gain of invasive growth as the ratio between the number of hyphal cells reached after 1,000 iterations of the model with and without resource allocation and division of labor (Fig. 4 *B* and *C*, color scale). For a given host susceptibility, the gain of growth increased with the level of resource allocation (*y* axis). In the absence of division of labor (Fig. 4*B*), resource allocation conferred a significant gain on susceptible hosts, and there was no growth on hosts with susceptibility <0.3. Division of labor extended fungal host range by enabling growth on very resistant hosts (susceptibility <0.3) that would otherwise not be colonized (Fig. 4*C*). On resistant hosts, division of labor and resource allocation operated in synergy to provide high fitness gain. In *S. sclerotiorum* GEM, C fluxes toward virulence calculated during *A. thaliana* colonization corresponded to a level of susceptibility of 0.21. We estimated a 38 to 45% growth reduction when apex cells were disconnected from the center cells (Fig. 3*A* and *SI Appendix*, Fig. S3), corresponding to a resource allocation level of ∼0.6. Based on our multicell model, we predict that division of labor was essential to allow *A. thaliana* colonization by *S. sclerotiorum* (Fig. 4*C*). A major prediction from the model is that the gain in fungal invasive growth conferred by cooperation is generally higher on resistant hosts than on susceptible hosts. In agreement, we found that the host plant *Nicotiana benthamiana* is more susceptible to *S. sclerotiorum* than *A. thaliana* (fungal growth rate 0.6 mm⋅h^−1^; *SI Appendix*, Fig. S3), and that ablation of central cells caused a stronger fungal growth penalty on *A. thaliana* (∼44.9%) than on *N. benthamiana* (∼19.4%, *P* = 0.007; *SI Appendix*, Fig. S3). We conclude that at the hyphae level the benefit from intercellular cooperation increases with the rate of resource allocation and the level of host resistance.

## Discussion

Recent discoveries have considerably expanded the repertoire of molecular determinants known to underlie microbial virulence at the cellular level (42, 43). For instance, fungal pathogens secrete up to hundreds of effector proteins and small metabolites to facilitate host colonization (44). Several reports suggest that effector secretion occurs locally in invasive hyphae (45, 46). However, despite frequent spatial heterogeneity of disease symptoms, evidence for a role of filamentous pathogen multicellular organization in virulence is lacking. Using systems biology and modeling approaches, our analyses demonstrate how local transcriptional programs in fungal cells favor the cooperative growth of multicellular hyphae in hostile host environments. Complex processes such as division of labor require mathematical modeling to generalize conclusions drawn from experiments (47, 48). Our study provides a framework combining experimental and modeling approaches to test for the adaptive significance of division of labor in many diseases caused by filamentous microbes. Fungal processes contributing to intercellular cooperation could also be exploited as novel targets to control diseases.

The division of tasks among specialized group members is an important aspect of major transitions in evolution that occurred many times independently (49, 50). Despite this, the evolutionary and ecological drivers leading to the establishment of division of labor remains an active area of research. Modeling approaches have identified several factors favorable to the evolution of division of labor and cooperation. At the population level, genetic relatedness, reciprocity, and group selection can lead to the emergence of cooperation (8). At the organism or multicellular level, the position of modules within an organism, existence of a fitness trade-off between two tasks, and synergistic interaction between modules favor division of labor (20). An experimental prisoner’s dilemma game in synthetic budding yeast colonies further demonstrated that spatial expansion is sufficient to drive enrichment in cooperators (22). Our analyses support the view that “colony growth alone” (14, 22), and the heterogeneity it creates in the environment, can promote cooperation in microbes. Pathogenic interactions with diverse hosts, increasing the range of environment encountered at the population level, could have favored the evolution of division of labor in fungi (13, 14). Synergistic interaction between metabolic pathways was shown to be favored when a pathway is of high metabolic burden or toxicity, highly complex, or relies on numerous extracellular steps (51). Several of these criteria are fulfilled in our study: *S. sclerotiorum* virulence involves the biosynthesis of complex toxins and secondary metabolites and the degradation of plant cell wall involves multiple extracellular enzymes (26, 52). In a previous study, we reported that fungal pathogens with a broad host range secrete more numerous and complex proteins than specialized fungi, likely increasing the metabolic burden of virulence in these species (53). These observations suggest that selection for the emergence of division of labor would be higher in broad-host-range fungi and may be related to fungal lifestyles.

Pathogens’ ability to colonize a host and trigger disease is determined largely by the virulence factors they express (54). The biosynthesis of virulence factors and their delivery to their site of action imposes a metabolic burden to pathogen cells. The existence of a trade-off between virulence and transmission, or virulence and within-host growth, is central to many theories on the evolution of parasites (55, 56). Trade-offs between virulence and transmission are also apparent in a growing number of experimental studies (57–59). Our results points toward division of labor as a strategy to mitigate virulence–transmission trade-offs in fungal pathogens through the optimization of resource usage and dampening toxicity of some fungal virulence factors and host defense compounds. The requirement for virulence factor biosynthesis comes in addition to the need for pathogen cells to grow and multiply to outcompete cooccurring microbes and disseminate efficiently. Cooccurring microbes and defector mutants may indeed benefit from secreted virulence factors produced by virulent pathogens manipulating host physiology in a “public-good” manner (60–62). In pathogenic public-good producers, reducing virulence, and thereby the ability to benefit from cooperation, may switch selection toward favoring cheats at high microbial density (63). The uneven distribution of nutrients made available by fungal pathogens may affect differently the growth of microbes forming the leaf microbiome. Consequently, disease management strategies exploiting virulence reduction may have undesired effects due to the cooperative nature of fungal virulence. Indeed, weakly virulent strains can alleviate rate–efficiency trade-offs on resource use and result in higher prevalence of virulent strains in natural populations (62). In addition, the silencing of secreted virulence factors may prove inefficient due to compensation through cooperative hyphal organization, increased natural selection on cooperative resource allocation, and resistance to host defense compounds, ultimately displacing natural population balance toward more aggressive strains.

## Materials and Methods

### Plant and Fungus Material and Growth Conditions.

*A. thaliana* plants of accession Col-0 were grown in Jiffy pots at 22 °C, with a 9-h light period under a light intensity of 120 μmol⋅m^−2^⋅s^−1^ for 4 wk before infection. *S. sclerotiorum* strain 1980 was subcultured on potato dextrose agar plates at 22 °C. For inoculations, a 5-mm-wide agar plug containing actively growing *S. sclerotiorum* mycelium was placed on the adaxial surface of leaves and plants were maintained at 80% humidity in Percival AR-41L3 chambers under the same day/light condition as for plant growth, in trays closed with plastic wrap to control for humidity.

### Transcriptomic Analyses.

*S. sclerotiorum* gene expression was analyzed in six growth conditions: (*i*) mycelium cultured in potato dextrose broth (PDB) as described in ref. 64, (*ii*) mycelium grown on minimum agar medium as described in ref. 64, (*iii* and *iv*) the center and margin of mycelium (colony diameter 25 mm) on potato dextrose agar plates, and (*v* and *vi*) the center and margin of disease lesion (lesion diameter 25 mm) on *A. thaliana* (Col-0 genotype). Samples were collected in three independent biological experiments. RNA extraction, sequencing, and reads mapping were performed as described in ref. 64. Normalized read count per gene and differential gene expression (relative to PDB-grown fungus) were calculated using the Bioconductor/DESeq2 (version 1.8.2) package. Raw and normalized RNA-seq data have been deposited in the GEO database (accession nos. GSE106811 and GSE116194). Genes were considered significantly up-regulated when induced fourfold or greater compared with the control PDB condition, with a *P* value ≤ 0.01 (Dataset S1). Normalized read counts per gene for the top 500 most-expressed genes were shuffled between the three biological replicates 100 times for principal component analysis (PCA). PCA was performed using the factomineR and factoextra R packages. For each PFAM domain, the number of differentially expressed genes was compared with the total number of genes in *S. sclerotiorum* v2 genome (52) using a χ^2^ test implemented in R to determine enrichment *P* values.

### Quantitative Real-Time PCR Analysis.

RNA extraction was performed as described above. First-strand cDNA synthesis was performed using 1 µg of total RNA with an anchored oligo(dT) and the SuperScript III reverse transcriptase (Invitrogen), according to the manufacturer’s instructions. Real-time PCR reactions were performed on a Light Cycler 480 thermocycler (Roche) at 60 °C annealing temperature using 1:10 diluted cDNAs and LightCycler 480 SYBR Green I Master (Roche) in a final reaction volume of 7 µL with primer pairs given in *SI Appendix*, Table S1. Transcript levels were normalized to both Sscle02g015170 and Sscle05g041680 reference gene transcript levels. The average of three independent biological replicates ± SD is shown.

### Ablation Assays and Linear Hyphal Growth Measurements.

Detached leaves of 4-wk-old *A. thaliana* plants (Col-0 accession) and *N. benthamiana* plants were inoculated with a 3-mm-wide agar plug containing actively growing *S. sclerotiorum* 1980. Leaves were maintained on wet paper towel in Petri dishes for the duration of the experiment. Pictures were taken every 30 min between 10 and 20 hpi. At 13 hpi, a circular area colonized by the fungus of 3- to 4-mm radius was removed with a scalpel from a subset of leaves (circular ablation). To control for wounding effects, transverse cuts through the colonized area or in distal parts of the leaves were applied with a scalpel to another subset of leaves (transverse and distal cuts). For each leaf and at each time point, the radius of colonized area was measured along four orthogonal directions with ImageJ version 1.51. This assay was repeated three times with similar results. In another set of experiments, at 13 hpi we removed a semicircular area colonized by the fungus of 2- to 3-mm radius (semicircular ablation) or made a semicircular cut. The radius of colonized area was measured along three directions at 45° angle on the half lesion containing the ablation/cut as well as three directions on the untouched half lesion (*SI Appendix*, Fig. S2). This assay was repeated twice with similar results. The radial hyphal growth rate *in planta* was computed as the slope of the linear regression along measured radii. Growth rates were calculated during the 9- to 13-hpi interval and during the 13- to 17-hpi interval.

### GEM Reconstruction and FBA.

A draft model for *S. sclerotiorum* 1980 was obtained first by transfer from already reconstructed models of the Ascomycete fungi *Saccharomyces cerevisiae* S288C (65) and *Aspergillus niger* CBS 513.88 (66) based on gene homology; see Peyraud et al. (58) for detailed algorithm. Briefly, reactions from available models including genes with orthologs (>30% identity and >50% coverage) in *S. sclerotiorum* 1980 genome (52) were collected. Gene orthology was further assessed using Inparanoid (67). The identifiers for these reactions were standardized using SAMIR (Semi Automatic Metabolic Identifier Reconciliation) (58) and identical reactions from each model were merged into a single one. Then, manual curation of the model (68) yielded a high-quality genome-scale model. Dead-end reactions were identified using MetExplore (69) followed by a manual gap-filling step. Transport reactions and assimilation pathways were curated based on Biolog phenotype microarrays analysis. Stoichiometry-balanced cycles were identified by FBA temporarily excluding open exchange fluxes and then were curated. The macromolecule secretion module (macromolecule biosynthesis and secretion) was reconstructed based on genomic predictions for protein secretion using WoLF PSORT (70) and SignalP4.0 (71), in addition to secreted macromolecules previously reported in the literature (Dataset S4). The cost of secretion was assigned by assuming one ATP hydrolyzed per metabolite unit transported through the fungal plasma membrane when no information was available. The plant cell-wall degradation module was modeled as hydrolysis reactions of plant cell-wall macromolecules. Generic macromolecule complexes describing the repetitive structural and chemical motif of the different plant cell-wall components were designed. Reactions cleaving these components were identified manually from plant cell-wall-degrading enzymes annotated in the genome (52). The model was converted to Systems Biology Markup Language (SBML) format following the MIRIAM Registry recommendations (Dataset S2). To quantitatively validate the model, we measured *S. sclerotiorum* growth kinetics in liquid minimal medium containing 50 mM d-glucose as the only carbon source and determined cell dry weight, total protein concentration, and substrate uptake rate by NMR as described in Peyraud et al. (58) (Dataset S5). Published reports on various Ascomycete fungal species were used to derive *S. sclerotiorum* biomass composition (Dataset S6). Flux distributions through the model were determined by FBA using FlexFlux (32), optimizing for biomass production as the objective function. Exchange fluxes corresponding to substrates available *in planta* were constrained based largely on data from AraGEM (72). Substrate uptake rates were constrained from experimentally measured fluxes when available or the upper bound was fixed to 5 mmol⋅g (cell dry weight)^−1^⋅h^−1^ as default value. Fitting of the flux distribution with the transcriptomic data were performed using the FlexFluxOmics algorithm available at https://github.com/lmarmiesse/FlexFlux/tree/master/src/flexflux/omics. The relative flux distributions were normalized using experimentally measured growth rates.

### Phenotype Microarrays.

Phenotype microarrays analysis was performed using Biolog plates PM1-3. To produce homogeneous fractions of *S. sclerotiorum* mycelium, strain 1980 was grown in PDB at 24 °C for 4 d under 180-rpm shaking. The culture was blended for 10 s and filtered through sterile 100-µm filters on a vacuum pump. The flow-through was collected and filtered through sterile 40-µm filters. The 40-µm filter membranes were washed with PDB to resuspend and collect mycelium fragments between 40 and 100 µm long. These fragments were cultured in PDB for 24 h at 24 °C at 180 rpm then filtered on 100 µm. The filter membranes containing fragments that grew above 100 µm were washed with minimum medium with no carbon source, and the resulting mycelium suspension was diluted to 62% transmittance. d-glucose at 20 mM was added for the inoculation of plates PM3. Plates were inoculated following the manufacturer’s instructions with the following modifications: the volumes of mycelium suspensions used to obtain a final 24 mL of inoculation solution were 1.5 mL for PM1 and 2, 0.3 mL for PM9, and 3 mL for the other plates. Measurements were recorded on an Omnilog reader (Biolog) every 30 min for 7 d. Six biological replicates were run for each plate. The data were analyzed using the R package OPM (Dataset S3).

### Multicell Hyphae Model.

A coarse-grain model was designed to assess the spatiotemporal effect of resource allocation and division of labor. The hyphae were modeled as a growing cylinder composed of a row of cells of identical volume. Every cell of the hyphae produced virulence factors from carbon uptake. Turgor pressure in cells was assumed constant and did not constrain growth. Under these conditions, growth depends only on the speed of cell-wall biosynthesis (73, 74), which occurs only in the apical cell (40). In the same manner, the colonized host was modeled as a stack of cells of identical volume containing carbon. Under the latter assumptions, the net variation of carbon uptake for every cell ***i*** different from the apical one was

$$\frac{{\text{dN}}_{\text{fungus},\text{i}}}{\text{dt}}=\frac{{\text{dN}}_{\text{host}\to \text{fungus},\text{i}}}{\text{dt}}-\frac{{\text{dN}}_{\text{vf},\text{i}}}{\text{dt}}+\frac{{\text{dN}}_{\text{diff},\text{i}}}{\text{dt}},$$ [1]

whereas in the apical cell the production rate of growth factor was

$$\frac{{\text{dN}}_{\text{growth},\text{apical}}}{\text{dt}}=\frac{{\text{dN}}_{\text{host}\to \text{fungus},\text{apical}}}{\text{dt}}-\frac{{\text{dN}}_{\text{vf},\text{apical}}}{\text{dt}}+\frac{{\text{dN}}_{\text{diff},\text{apical}}}{\text{dt}},$$ [2]

with N_fungus,i_ the quantity of nutrients uptaken by the cell ***i***, N_vf,i_ the quantity of virulence factor produced by the cell ***i***, and N_growth,apical_ the quantity of cell wall polysaccharides produced by the apical cell. dN_host→fungus,i_/dt and dN_diff,i_/dt were the speeds at which carbon was extracted from host and exchanged with neighboring cell. They were modeled by Fick’s law:

$${\left(\frac{{\text{dN}}_{\text{uptake},\text{i}}}{\text{dt}}\right)}_{\text{host}\to \text{fungus},\text{i}}\;={\text{D}}_{\text{host}-\text{fungus}}\;\left({\text{N}}_{\text{host},\text{i}}-{\text{N}}_{\text{hyphae},\text{i}}\right)$$ [3]

$${\left(\frac{{\text{dN}}_{\text{diff},\text{i}}}{\text{dt}}\right)}_{\text{hyphae}}\;={\text{D}}_{\text{hyphae}-\text{hyphae}}\left({\text{N}}_{\text{hyphae},\text{i}}-{\text{N}}_{\text{hyphal},\text{i}-1}\right)$$ [4]

with D_host–fungus_ and D_hyphae–hyphae_ coefficients of diffusion (in [0,1/2]).

Growth of the hyphae was discretized: When carbon quantity in the apical cell overcame a threshold (N_growth_), a new cell was produced. The resistance of a host cell was assumed to vanish when a certain amount of virulence factor was secreted by the associated cell of the pathogen. When the resistance of a host cell vanished, nutrients were free to diffuse to the hyphae and the local metabolism of the hyphae switched from apical to central. On the basis of results provided by metabolic reconstruction, the rate of production of virulence factors was assumed proportional to the carbon uptake of the cell:

$$\frac{{\text{dN}}_{\text{vf},\text{i}}}{\text{dt}}=\mu_{\text{i}}\;\left(\frac{{\text{dN}}_{\text{host}\to \text{fungus},\text{i}}}{\text{dt}}+\frac{{\text{dN}}_{\text{diff},\text{i}}}{\text{dt}}\right).$$ [5]

The μ_i_ parameter had two values depending on the cell location (center or apex). The parameter μ was introduced to describe the bias in resources allocation in the production of virulence factors between center and apex of the colony:

$$\mu_{i\;\in \;\mathrm{center}}=\;\mu .\mu_{j\;\in \;\mathrm{periphery}}\mathrm{with}\;\mu <1.$$ [6]

### Simulation of Hyphal Invasive Growth.

We assumed an infinite quantity of carbon in the host cell which involved a constant speed of uptake (N_host,i_ >> N_hyphae,i_). On the basis of results provided by GEM analysis, we assumed constant uptake from host, identical for cells in center and in apex of the hyphae, and not dependent on host resistance. The speed of uptake was set to 1 mmol per simulation time unit. Increase in host resistance was modeled by the increase in the bias of resources allocation in the production of virulence factor μ. μ_i∈center_ was assumed constant and set to 0.03. Low host resistance was associated with μ = 1, whereas high host sensibility was associated with μ close to 0 (Eq. **6**). The level of resource allocation between cells was modeled by the dimensionless number

$$\alpha =D_{\mathrm{hyphae-hyphae}}/0.5\in \left[0,1\right].$$ [7]

The sensitivity of hyphal growth to resource allocation was tested by varying α. The sensitivity of hyphal growth to division of labor was tested by setting μ_i∈center_ to the same value as μ_j∈apex_. In every test set, simulations were initiated with hyphae composed of an apical cell. Simulations were computed over 1,000 iterations and length of the modeled hyphae was reported. Gain for a given host susceptibility S was computed as

$$\text{G}\left(\alpha \right){|}_{\text{S}}=\left(\frac{{\Delta \text{L}}_{\alpha \neq 0}}{{\Delta \text{L}}_{\alpha =0}}\right){|}_{\text{S}}\;-1=\left(\frac{{\text{L}}_{\alpha \neq 0}\left(\text{t}=1,000\right)-{\text{L}}_{\alpha \neq 0}\left(\text{t}=0\right)}{{\text{L}}_{\alpha =0}\left(\text{t}=1,000\right)-{\text{L}}_{\alpha =0}\left(\text{t}=0\right)}\right){|}_{\text{S}}-1.$$ [8]

The model implemented in Python 2.7 is available at https://github.com/QiPteam/intercellularcooperation.

### Data Availability.

Raw and normalized RNA-seq data have been deposited in the GEO database (accession nos. GSE106811 and GSE116194). Differentially expressed genes, GEM files, and phenotype microarray data are provided in Datasets S1–S6.

## Supplementary Material

Supplementary File

Supplementary File

Supplementary File

Supplementary File

Supplementary File

Supplementary File

Supplementary File
