# Supplementary material for: Intercellular cooperation in a fungal plant pathogen facilitates host colonization
Source: Proc Natl Acad Sci U S A. 2019 Feb 6;116(8):3193–201. doi: 10.1073/pnas.1811267116 (PMC6386666; doi:10.1073/pnas.1811267116)
Supplement: Supplementary File [file pnas.1811267116.sapp.pdf]

PNAS
www.pnas.org

Supplementary Information for

Intercellular cooperation in a fungal plant pathogen facilitates host colonization

Remi Peyraud, Malick Mbengue, Adelin Barbacci, Sylvain Raffaele

Correspondence: sylvain.raffaele@inra.fr

This PDF file includes:

Figure S1-S3
Table S1
Captions for datasets S1 to S6
Supplementary references

Other supplementary materials for this manuscript include the following:

Datasets S1 to S6

www.pnas.org/cgi/doi/10.1073/pnas.1811267116

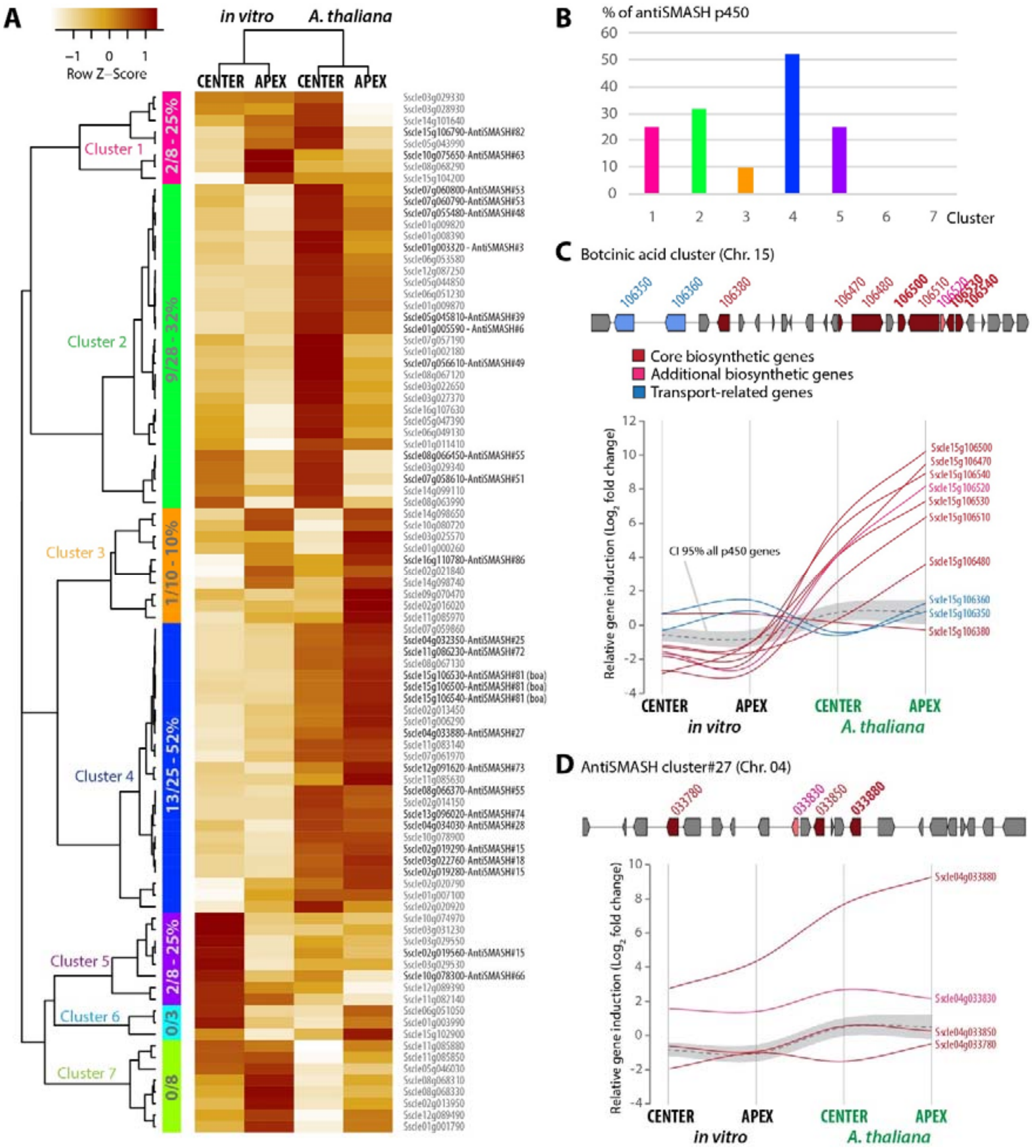

Fig. S1. Up-regulation of p450 genes associated with toxin biosynthesis at the apex of *S. sclerotiorum* hyphae. (A) Relative expression of the 90 genes encoding putative p450 cytochrome in the genome of *S. sclerotiorum* expressed both *in vitro* and *in planta*. We classified these genes based on their expression pattern using complete Pearson correlation with the hclust function in R, into 7 hierarchical clusters. To identifying those involved in toxin biosynthesis, we used the secondary metabolite analysis shell antiSMASH4.0 (1). This predicted 87 genomic clusters for the synthesis of secondary metabolites, including 22 clusters harboring a p450 gene. The percentage of genes in each expression cluster detected by antiSMASH is indicated in the color bars next to the hierarchical tree. Over 80% of p450 genes flagged by antiSMASH belonged to expression clusters 2 and 4 containing genes predominantly expressed *in planta*. **(B)** Percentage of genes detected by antiSMASH in each expression cluster. Notably,

52% of p450 genes from expression cluster 4, predominantly expressed at the apex *in planta*, were flagged by antiSMASH. **(C)** Expression of genes from antiSMASH cluster 81 responsible for the biosynthesis of toxin botcinic acid (boa). The median and 95% confidence interval for all p450 genes are shown as grey dotted line and grey area respectively. Only genes identified as part of the boa biosynthesis cluster by antiSMASH are shown, colored according to the function assigned by antiSMASH in agreement with Dalmais *et al*. (2). Genes encoding p450 cytochromes are labelled in bold. **(D)** Expression of genes from antiSMASH cluster 27 of uncharacterized function, including a p450 cytochrome. The same graphic codes as in (C) were used.

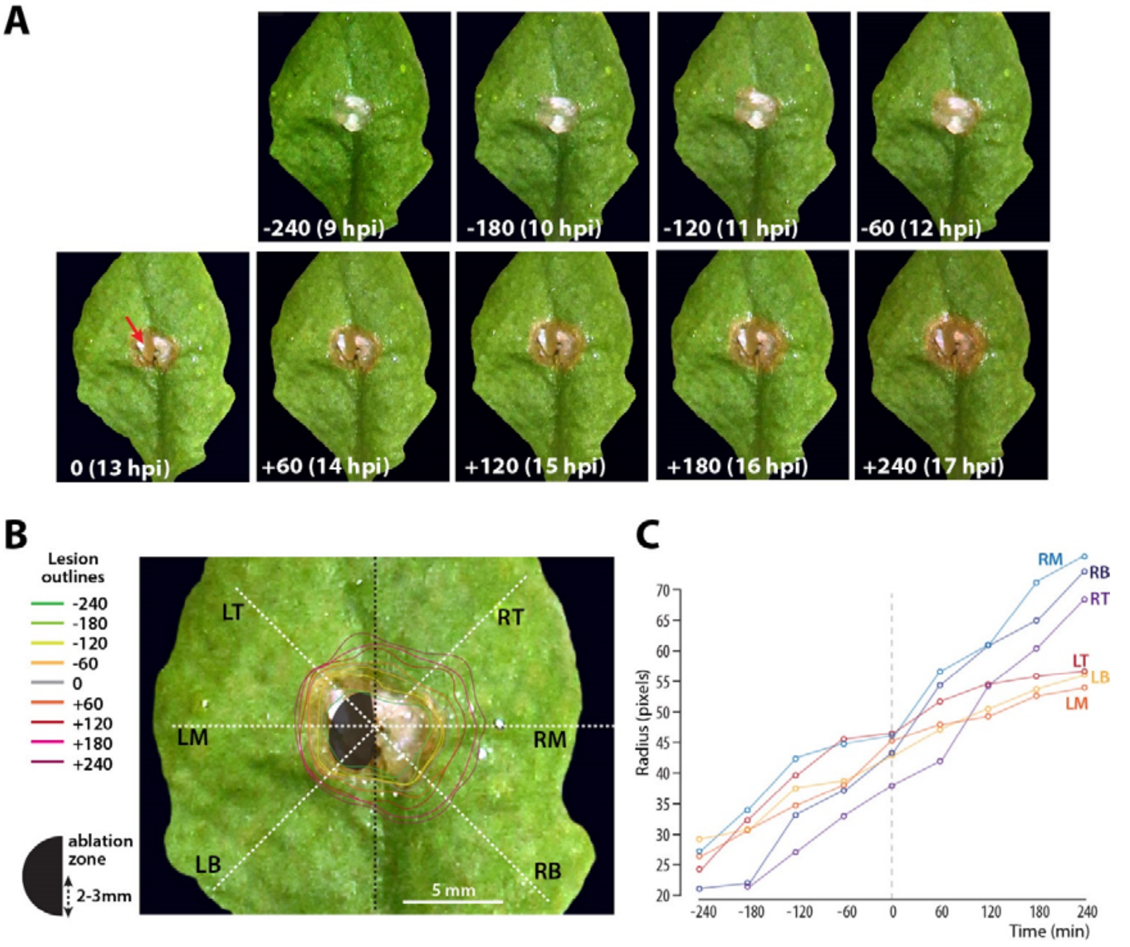

Fig. S2. Experimental test for the importance of fungal radial connectivity in disease lesion progression using semi-circular ablation of lesion center. (A) Representative symptoms on *A. thaliana* Col-0 plants from 9 to 17 hours post inoculation (hpi) by *S. sclerotiorum*. A semi-circular ablation was performed at 13 hpi on the left hand side of the lesion (red arrow). Images are labelled with time in minutes relative to the ablation. **(B)** Radial growth measurements on the same leaf as in (A). The outline of lesions at each time point (-240 to +240 minutes) is reported on a single composite image. The ablation zone, of radius 2-3 mm to leave ~1mm intact mycelium periphery, is shown a grey-shaded area. Lesion radii were measured along 6 directions indicated by dotted white lines and labelled with the following code: B, bottom; L, left; M, middle; R, right; T, top. **(C)** Measurements over time of lesion radii along the 6 directions shown in B. Radial growth speed was determined as the slope of these curves between time points -240 to 0 min (before treatment) and 0 to +240 min (after treatment). Hyphal tips on the left side of the lesion (radii LT, LM and LB) were disconnected from central cells and show a decreased in radial growth speed after ablation.

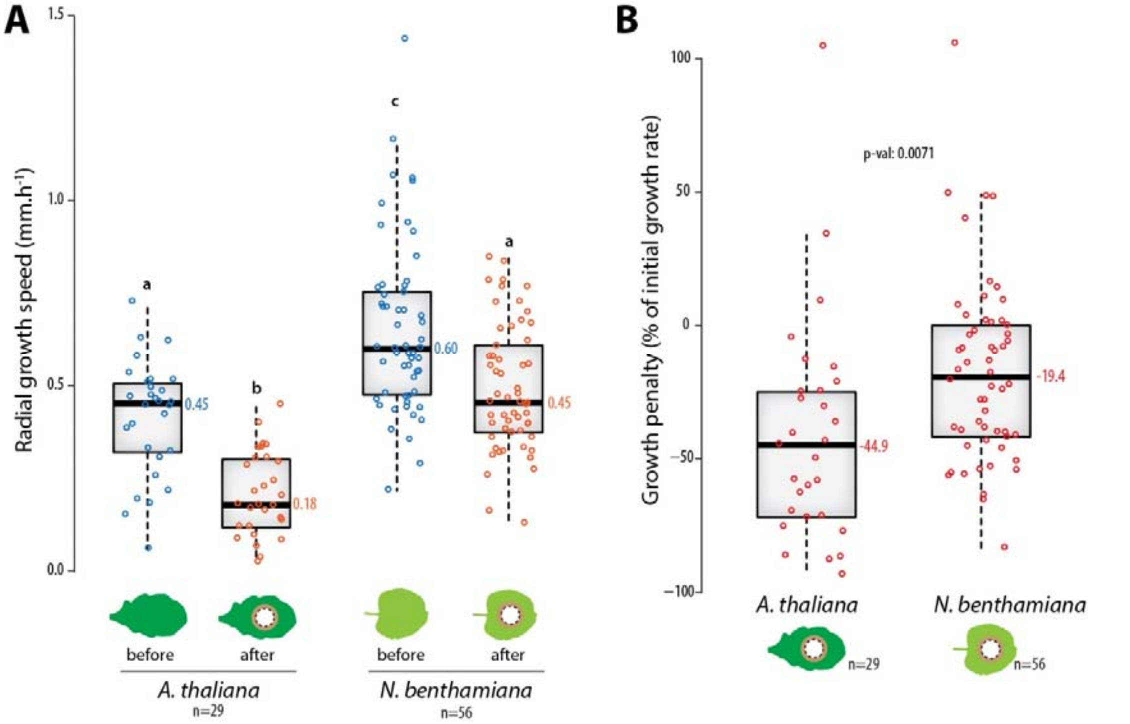

Fig. S3. *S. sclerotiorum* growth penalty caused by disruption of cooperation on a resistant (*A. thaliana*) and a susceptible (*N. benthamiana*) host. (A) Measure of S. sclerotiorum radial growth speed (mm. h^{-1}) on before (9 to 13 hours post-inoculation, hpi) and after (13 to 17 hpi) ablation of ~3-4mm central lesion area. Groups of significance were determined by pairwise Welch t tests (p-value <0.01). **(B)** Growth penalty after ablation determined as the percentage of initial growth rate from data shown in (A). Boxplots show 1st and 3rd quartiles (box), median (horizontal line, label) and the most dispersed values within 1.5 times the interquartile range (whiskers). Significance of the difference between hosts was assessed by a Welch t test.

Table S1: Oligonucleotide primers used in this work.

Target gene	Forward primer sequence	Reverse primer sequence
Sscle01g002960	5’-CAAGCAAATGCTGGTGGTTA-3’	5’-GGGGCGAATTTATCTTCAAA-3’
Sscle07g060710	5’-GCTGTTGTTCTGGTCAACGA-3’	5’-CAAAACCCGGTATCAGAGGA-3’
Sscle15g106490	5’-CTTCCTTGCTCTCGACAACC-3’	5’-GCGAGTTGTGTCTTTCACGA-3’
Sscle01g005560	5’-GCGTCGATTTCTACGCTTTC-3’	5’-TTTTTGGAGCGTTTCTTCGT-3’
Sscle08g068420	5’-GCAAGTAGCTTACCGGGTTG-3’	5’-CCACGGAACAATGCTTTACC-3’
Sscle16g107460	5’-CAGCTGTCAACCAAATCGAG-3’	5’-CTCCGGTGTAACGGATTTTG-3’
Sscle02g015170	5’-TTGGATTTGCTCCTTTGACC-3’	5’-GGGATCCACTCGACGAAGTA-3’
Sscle05g041680	5’-TTGGTGAAACTTCCCGAGTC-3’	5’-GGTATGAGGCGGTGTGAGAT-3’

Dataset S1. List of *S. sclerotiorum* differentially expressed genes (.xls workbook). A total of 1133 genes induced ≥ 4 folds either *in planta* or on solid medium *in vitro*. Only 53 genes were induced in all four conditions, and 218 genes were induced both *in vitro* and *in planta*. A total of 288 genes were induced *in vitro* only, and 627 were induced *in planta* only. A total of 224 genes were induced both at the center and at the apex of colonies. Among genes induced in the mycelium center only (306 in total), there was only 4 induced both *in vitro* and *in planta*. The file includes five tabs: 'LFC_complete' provides Log2 fold change in gene expression for all *S. sclerotiorum* genes calculated by R DESeq2 package; '1133_Induced_4x' is the list of the 1133 genes induced at least 4 fold in at least one condition. Subsets discussed in the main text are indicated in columns I to N; 'PFAM_annot' provides the complete PFAM annotation of *S. sclerotiorum* genes, including the corresponding gene expression values; 'PFAM_Xsquared' provides Chi-squared test for PFAM domain enrichment among genes induced *in vitro*, *in planta*, at the apex, at the center (p-values are not corrected) and 'Fig1E' provides raw data used for the generation of figure 1E, including PFAM enrichment p-values and cumulated RNAseq read counts.

Dataset S2. Genome-scale metabolic model of *S. sclerotiorum* in SBML format (.xml file).

Dataset S3. *S. sclerotiorum* growth on 288 metabolites determined using Biolog phenotype microarrays, compared to GEM predictions (.xls workbook). The file includes three tabs: 'Biolog results' provides raw experimental data of the Biolog phenotype microarrays experiments and statistical analysis performed using OPM R package; 'Simulation results' provides the results of flux balance analysis using *S. sclerotiorum* GEM; 'Analysis' provides the comparison between experimental and simulated data including network predictions performance values.

Dataset S4. Results of flux balance analysis in *S. sclerotiorum* GEM during *A. thaliana* colonization and *in vitro* growth (.xls workbook). 592 to 610 reactions supported non-null carbon fluxes in *S. sclerotiorum* cells, covering 101 to 103 distinct metabolic pathways. The file includes five tabs: 'Model_rxn', List of all reactions included in *S. sclerotiorum* GEM, including associated genes; 'Raw_FBA', Raw output of flux balance analysis in FlexFlux with *S. sclerotiorum* GEM for the 12 RNAseq samples (3 replicates per condition); 'Normalized_Fluxes' Metabolic fluxes normalized based on biomass production reaction; 'per_pathway' Sum of fluxes through reactions of a same metabolic pathway; 'select_Fig2C', Selection of pathways harboring significantly different fluxes in apex and center cells.

Dataset S5. Estimation of *S. sclerotiorum* growth kinetics parameters *in vitro*. The file includes three tabs: 'CellDryWeight', Relationship between mycelium suspension OD600nm and *S. sclerotiorum* 1980 cell dry weight; 'Kinetics_MMGlu', Measurement of *S. sclerotiorum* 1980 growth rate on minimal medium with 50mM D-Glucose as only carbon source; 'Kinetics_PDB', Measurement of *S. sclerotiorum* 1980 growth rate on Potato Dextrose Broth.

Dataset S6. *S.sclerotiorum* biomass composition (.xls workbook). The file includes two tabs: 'Biomass composition' provides biomass composition for *Sclerotinia sclerotiorum* used in the model iRP17119; 'Energetic' provides ATP maintenance cost calculation used in the model iRP17119.

References

1. Blin K, et al. (2017) antiSMASH 4.0-improvements in chemistry prediction and gene cluster boundary identification. *Nucleic Acids Res* 45(W1):W36–W41.

2. Dalmais B, et al. (2011) The Botrytis cinerea phytotoxin botcinic acid requires two polyketide synthases for production and has a redundant role in virulence with botrydial. *Mol Plant Pathol* 12(6):564–579.
